# Cytotoxicity of *Agave sisalana *in hemocytes of the mosquito *Aedes aegypti*, which transmits dengue

**DOI:** 10.1186/1753-6561-8-S4-P60

**Published:** 2014-10-01

**Authors:** Débora Lacerda, Louise Oliveira, Fabiola Nunes

**Affiliations:** 1 Biotechnology Center, Federal University of Paraiba, João Pessoa, Paraíba, 58037760, Brazil

## Background

The dengue is a viral disease transmitted by a mosquito from the Culicidae family from Africa, the *Aedes aegypti*. Most common in tropical and subtropical regions, this mosquito has diurnal activity and is difficult to control due to the high resistance of its eggs and its quick transformation into larvae. The severe illness may cause death, most commonly presenting symptoms such as high fever, pain behind the eyes and headaches.

## Methods

Fifteen larvae (L4) were exposed in a 6.5 mg/mL concentration of *A.sisalana *juice, during the periods of 3, 6, 12 and 24 hours, completing 4 experimental groups and one control group for each, with means, 4 control groups.

## Results and conclusions

The A. sisalana had a cytotoxic necrotizing effect on *A. aegypti *hemocytes, showing that after 12 hours of exposition the larvae in juice had a 21% hemocytes mortality compared with the control group. After 24 hours, a cell mortality of 16.5% in relation with the control group, as well the growth of hemocytes' size and granularity, being compatible with the noted necrosis. In fact, this effect was acute, because, after 24 hours, the majority of larvae were dead even when a sub-lethal concentration was used, what suggest that this is the major sisal juice action mechanism.

